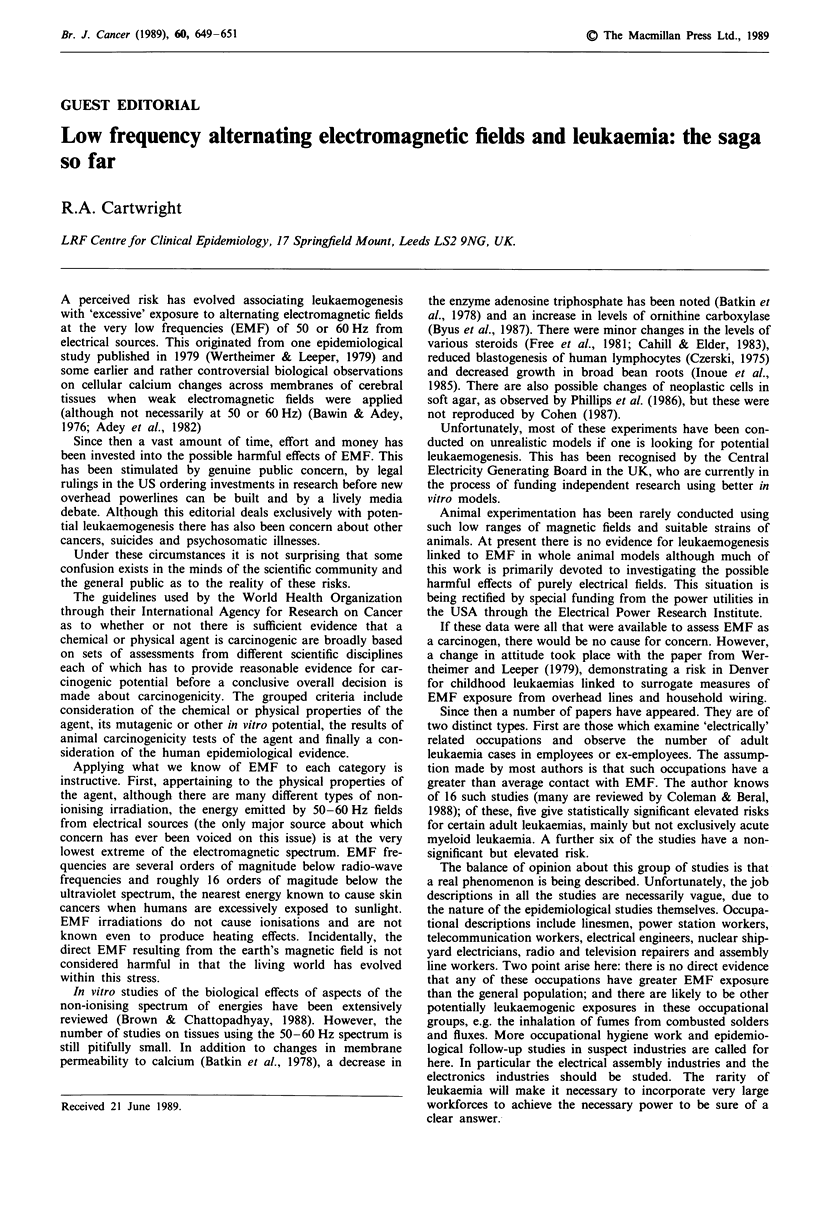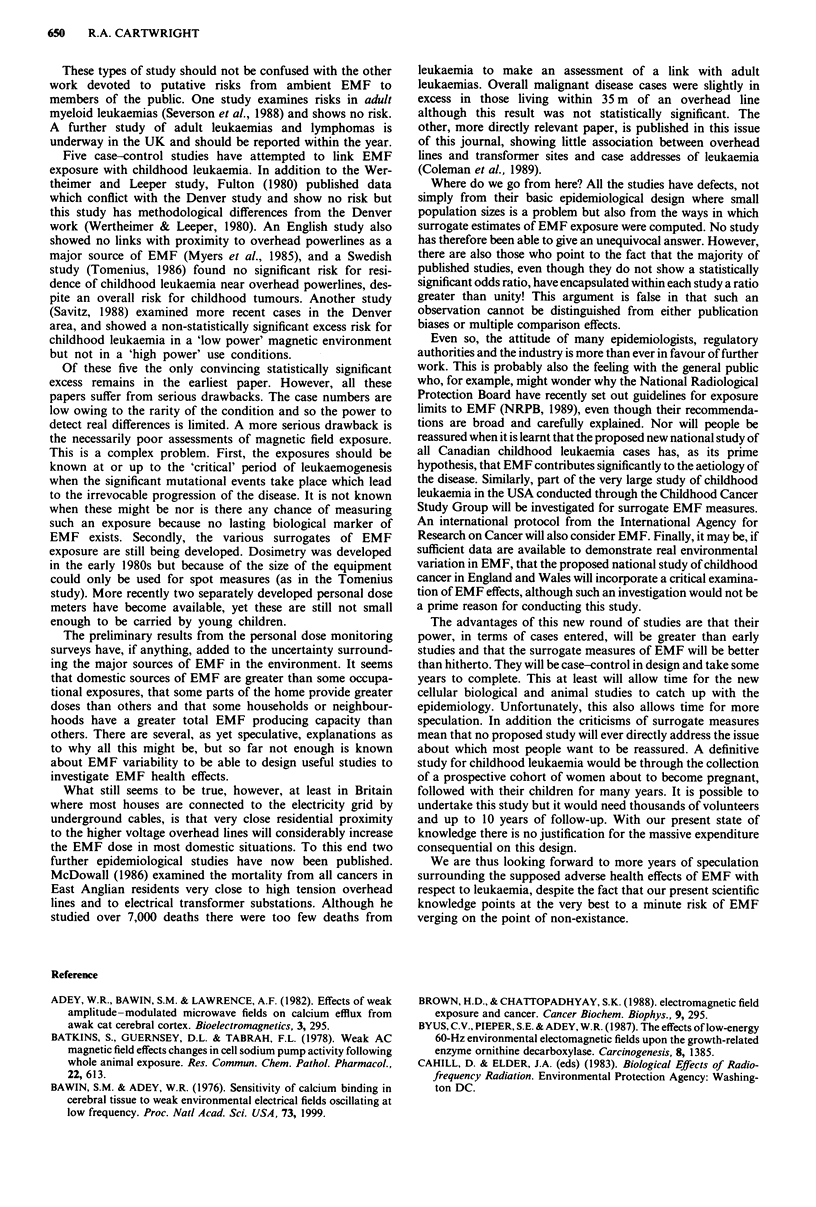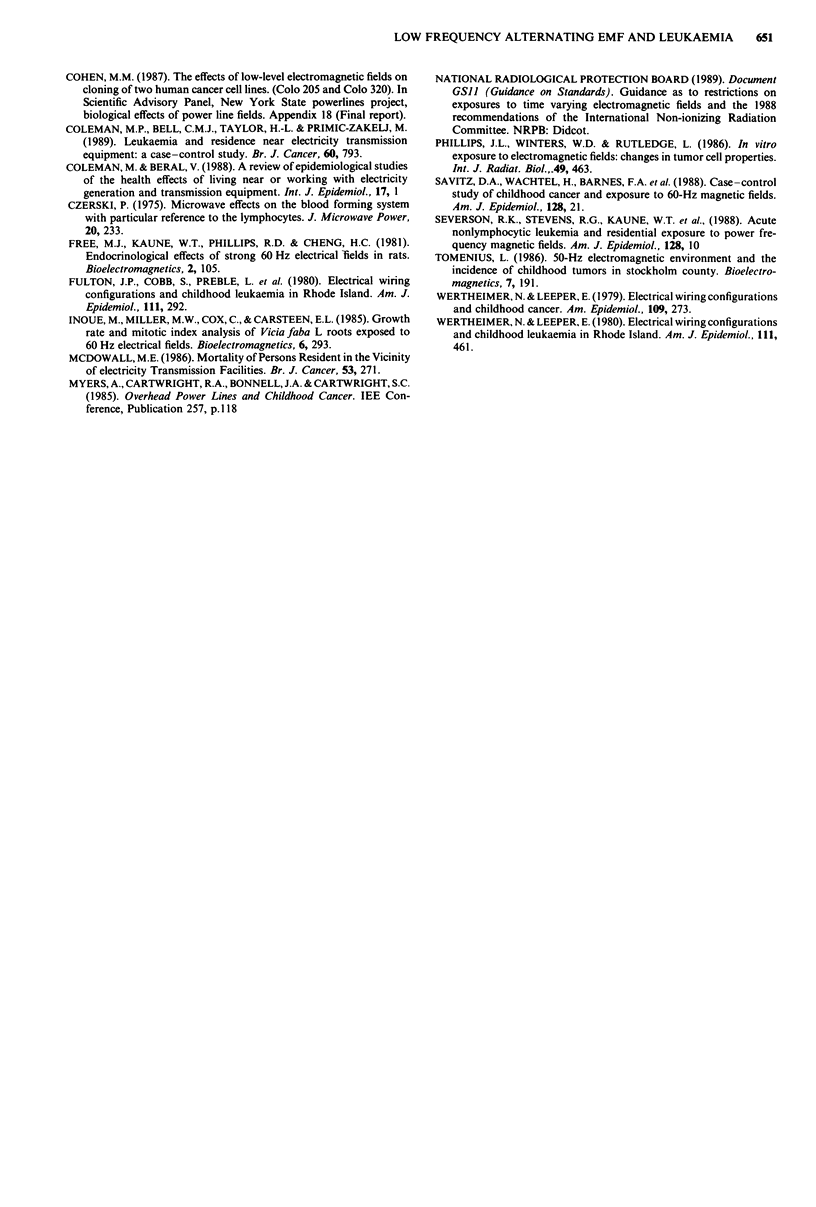# Low frequency alternating electromagnetic fields and leukaemia: the saga so far.

**DOI:** 10.1038/bjc.1989.332

**Published:** 1989-11

**Authors:** R. A. Cartwright

**Affiliations:** LRF Centre for Clinical Epidemiology, Leeds, UK.


Br. J. Cancer (1989), 60, 649–651 © The Macmillan Press Ltd., 1989

GUEST EDITORIAL

### Low frequency alternating electromagnetic fields and leukaemia: the saga so far

R.A. Cartwright

*LRF Centre for Clinical Epidemiology, 17 Springfield Mount, Leeds LS2 9NG, UK.*

A perceived risk has evolved associating leukaemogenesis with 'excessive' exposure to alternating electromagnetic fields at the very low frequencies (EMF) of 50 or 60 Hz from electrical sources. This originated from one epidemiological study published in 1979 (Wertheimer & Leeper, 1979) and some earlier and rather controversial biological observations on cellular calcium changes across membranes of cerebral tissues when weak electromagnetic fields were applied (although not necessarily at 50 or 60 Hz) (Bawin & Adey, 1976; Adey *et al.*, 1982)

Since then a vast amount of time, effort and money has been invested into the possible harmful effects of EMF. This has been stimulated by genuine public concern, by legal rulings in the US ordering investments in research before new overhead powerlines can be built and by a lively media debate. Although this editorial deals exclusively with potential leukaemogenesis there has also been concern about other cancers, suicides and psychosomatic illnesses.

Under these circumstances it is not surprising that some confusion exists in the minds of the scientific community and the general public as to the reality of these risks.

The guidelines used by the World Health Organization through their International Agency for Research on Cancer as to whether or not there is sufficient evidence that a chemical or physical agent is carcinogenic are broadly based on sets of assessments from different scientific disciplines each of which has to provide reasonable evidence for carcinogenic potential before a conclusive overall decision is made about carcinogenicity. The grouped criteria include consideration of the chemical or physical properties of the agent, its mutagenic or other *in vitro* potential, the results of animal carcinogenicity tests of the agent and finally a consideration of the human epidemiological evidence.

Applying what we know of EMF to each category is instructive. First, appertaining to the physical properties of the agent, although there are many different types of non-ionising irradiation, the energy emitted by 50–60 Hz fields from electrical sources (the only major source about which concern has ever been voiced on this issue) is at the very lowest extreme of the electromagnetic spectrum. EMF frequencies are several orders of magnitude below radio-wave frequencies and roughly 16 orders of magitude below the ultraviolet spectrum, the nearest energy known to cause skin cancers when humans are excessively exposed to sunlight. EMF irradiations do not cause ionisations and are not known even to produce heating effects. Incidentally, the direct EMF resulting from the earth's magnetic field is not considered harmful in that the living world has evolved within this stress.

*In vitro* studies of the biological effects of aspects of the non-ionising spectrum of energies have been extensively reviewed (Brown & Chattopadhyay, 1988). However, the number of studies on tissues using the 50–60 Hz spectrum is still pitifully small. In addition to changes in membrane permeability to calcium (Batkin *et al.*, 1978), a decrease in the enzyme adenosine triphosphate has been noted (Batkin *et al.*, 1978) and an increase in levels of ornithine carboxylase (Byus *et al.*, 1987). There were minor changes in the levels of various steroids (Free *et al.*, 1981; Cahill & Elder, 1983), reduced blastogenesis of human lymphocytes (Czerski, 1975) and decreased growth in broad bean roots (Inoue *et al.*, 1985). There are also possible changes of neoplastic cells in soft agar, as observed by Phillips *et al.* (1986), but these were not reproduced by Cohen (1987).

Unfortunately, most of these experiments have been conducted on unrealistic models if one is looking for potential leukaemogenesis. This has been recognised by the Central Electricity Generating Board in the UK, who are currently in the process of funding independent research using better *in vitro* models.

Animal experimentation has been rarely conducted using such low ranges of magnetic fields and suitable strains of animals. At present there is no evidence for leukaemogenesis linked to EMF in whole animal models although much of this work is primarily devoted to investigating the possible harmful effects of purely electrical fields. This situation is being rectified by special funding from the power utilities in the USA through the Electrical Power Research Institute.

If these data were all that were available to assess EMF as a carcinogen, there would be no cause for concern. However, a change in attitude took place with the paper from Wertheimer and Leeper (1979), demonstrating a risk in Denver for childhood leukaemias linked to surrogate measures of EMF exposure from overhead lines and household wiring.

Since then a number of papers have appeared. They are of two distinct types. First are those which examine 'electrically' related occupations and observe the number of adult leukaemia cases in employees or ex-employees. The assumption made by most authors is that such occupations have a greater than average contact with EMF. The author knows of 16 such studies (many are reviewed by Coleman & Beral, 1988); of these, five give statistically significant elevated risks for certain adult leukaemias, mainly but not exclusively acute myeloid leukaemia. A further six of the studies have a non-significant but elevated risk.

The balance of opinion about this group of studies is that a real phenomenon is being described. Unfortunately, the job descriptions in all the studies are necessarily vague, due to the nature of the epidemiological studies themselves. Occupational descriptions include linesmen, power station workers, telecommunication workers, electrical engineers, nuclear shipyard electricians, radio and television repairers and assembly line workers. Two point arise here: there is no direct evidence that any of these occupations have greater EMF exposure than the general population; and there are likely to be other potentially leukaemogenic exposures in these occupational groups, e.g. the inhalation of fumes from combusted solders and fluxes. More occupational hygiene work and epidemiological follow-up studies in suspect industries are called for here. In particular the electrical assembly industries and the electronics industries should be studed. The rarity of leukaemia will make it necessary to incorporate very large workforces to achieve the necessary power to be sure of a clear answer.

Received 21 June 1989.

These types of study should not be confused with the other work devoted to putative risks from ambient EMF to members of the public. One study examines risks in *adult* myeloid leukaemias (Severson *et al.*, 1988) and shows no risk. A further study of adult leukaemias and lymphomas is underway in the UK and should be reported within the year.

Five case-control studies have attempted to link EMF exposure with childhood leukaemia. In addition to the Wertheimer and Leeper study, Fulton (1980) published data which conflict with the Denver study and show no risk but this study has methodological differences from the Denver work (Wertheimer & Leeper, 1980). An English study also showed no links with proximity to overhead powerlines as a major source of EMF (Myers *et al.*, 1985), and a Swedish study (Tomenius, 1986) found no significant risk for residence of childhood leukaemia near overhead powerlines, despite an overall risk for childhood tumours. Another study (Savitz, 1988) examined more recent cases in the Denver area, and showed a non-statistically significant excess risk for childhood leukaemia in a 'low power' magnetic environment but not in a 'high power' use conditions.

Of these five the only convincing statistically significant excess remains in the earliest paper. However, all these papers suffer from serious drawbacks. The case numbers are low owing to the rarity of the condition and so the power to detect real differences is limited. A more serious drawback is the necessarily poor assessments of magnetic field exposure. This is a complex problem. First, the exposures should be known at or up to the 'critical' period of leukaemogenesis when the significant mutational events take place which lead to the irrevocable progression of the disease. It is not known when these might be nor is there any chance of measuring such an exposure because no lasting biological marker of EMF exists. Secondly, the various surrogates of EMF exposure are still being developed. Dosimetry was developed in the early 1980s but because of the size of the equipment could only be used for spot measures (as in the Tomenius study). More recently two separately developed personal dose meters have become available, yet these are still not small enough to be carried by young children.

The preliminary results from the personal dose monitoring surveys have, if anything, added to the uncertainty surrounding the major sources of EMF in the environment. It seems that domestic sources of EMF are greater than some occupational exposures, that some parts of the home provide greater doses than others and that some households or neighbourhoods have a greater total EMF producing capacity than others. There are several, as yet speculative, explanations as to why all this might be, but so far not enough is known about EMF variability to be able to design useful studies to investigate EMF health effects.

What still seems to be true, however, at least in Britain where most houses are connected to the electricity grid by underground cables, is that very close residential proximity to the higher voltage overhead lines will considerably increase the EMF dose in most domestic situations. To this end two further epidemiological studies have now been published. McDowall (1986) examined the mortality from all cancers in East Anglian residents very close to high tension overhead lines and to electrical transformer substations. Although he studied over 7,000 deaths there were too few deaths from leukaemia to make an assessment of a link with adult leukaemias. Overall malignant disease cases were slightly in excess in those living within 35 m of an overhead line although this result was not statistically significant. The other, more directly relevant paper, is published in this issue of this journal, showing little association between overhead lines and transformer sites and case addresses of leukaemia (Coleman *et al.*, 1989).

Where do we go from here? All the studies have defects, not simply from their basic epidemiological design where small population sizes is a problem but also from the ways in which surrogate estimates of EMF exposure were computed. No study has therefore been able to give an unequivocal answer. However, there are also those who point to the fact that the majority of published studies, even though they do not show a statistically significant odds ratio, have encapsulated within each study a ratio greater than unity! This argument is false in that such an observation cannot be distinguished from either publication biases or multiple comparison effects.

Even so, the attitude of many epidemiologists, regulatory authorities and the industry is more than ever in favour of further work. This is probably also the feeling with the general public who, for example, might wonder why the National Radiological Protection Board have recently set out guidelines for exposure limits to EMF (NRPB, 1989), even though their recommendations are broad and carefully explained. Nor will people be reassured when it is learnt that the proposed new national study of all Canadian childhood leukaemia cases has, as its prime hypothesis, that EMF contributes significantly to the aetiology of the disease. Similarly, part of the very large study of childhood leukaemia in the USA conducted through the Childhood Cancer Study Group will be investigated for surrogate EMF measures. An international protocol from the International Agency for Research on Cancer will also consider EMF. Finally, it may be, if sufficient data are available to demonstrate real environmental variation in EMF, that the proposed national study of childhood cancer in England and Wales will incorporate a critical examination of EMF effects, although such an investigation would not be a prime reason for conducting this study.

The advantages of this new round of studies are that their power, in terms of cases entered, will be greater than early studies and that the surrogate measures of EMF will be better than hitherto. They will be case-control in design and take some years to complete. This at least will allow time for the new cellular biological and animal studies to catch up with the epidemiology. Unfortunately, this also allows time for more speculation. In addition the criticisms of surrogate measures mean that no proposed study will ever directly address the issue about which most people want to be reassured. A definitive study for childhood leukaemia would be through the collection of a prospective cohort of women about to become pregnant, followed with their children for many years. It is possible to undertake this study but it would need thousands of volunteers and up to 10 years of follow-up. With our present state of knowledge there is no justification for the massive expenditure consequential on this design.

We are thus looking forward to more years of speculation surrounding the supposed adverse health effects of EMF with respect to leukaemia, despite the fact that our present scientific knowledge points at the very best to a minute risk of EMF verging on the point of non-existance.

### Reference

ADEY, W.R., BAWIN, S.M. & LAWRENCE, A.F. (1982). Effects of weak amplitude-modulated microwave fields on calcium efflux from awak cat cerebral cortex. *Bioelectromagnetics*, **3**, 295.

BATKINS, S., GUERNSEY, D.L. & TABRAH, F.L. (1978). Weak AC magnetic field effects changes in cell sodium pump activity following whole animal exposure. *Res. Commun. Chem. Pathol. Pharmacol.*, **22**, 613.

BAWIN, S.M. & ADEY, W.R. (1976). Sensitivity of calcium binding in cerebral tissue to weak environmental electrical fields oscillating at low frequency. *Proc. Natl Acad. Sci. USA*, **73**, 1999.

BROWN, H.D., & CHATTOPADHYAY, S.K. (1988). electromagnetic field exposure and cancer. *Cancer Biochem. Biophys.*, **9**, 295.

BYUS, C.V., PIEPER, S.E. & ADEY, W.R. (1987). The effects of low-energy 60-Hz environmental electomagnetic fields upon the growth-related enzyme ornithine decarboxylase. *Carcinogenesis*, **8**, 1385.

CAHILL, D. & ELDER, J.A. (eds) (1983). *Biological Effects of Radiofrequency Radiation.* Environmental Protection Agency: Washington DC.

COHEN, M.M. (1987). The effects of low-level electromagnetic fields on cloning of two human cancer cell lines. (Colo 205 and Colo 320). In Scientific Advisory Panel, New York State powerlines project, biological effects of power line fields. Appendix 18 (Final report).

COLEMAN, M.P., BELL, C.M.J., TAYLOR, H.-L. & PRIMIC-ZAKELJ, M. (1989). Leukaemia and residence near electricity transmission equipment: a case-control study. *Br. J. Cancer*, **60**, 793.

COLEMAN, M. & BERAL, V. (1988). A review of epidemiological studies of the health effects of living near or working with electricity generation and transmission equipment. *Int. J. Epidemiol.*, **17**, 1

CZERSKI, P. (1975). Microwave effects on the blood forming system with particular reference to the lymphocytes. *J. Microwave Power*, **20**, 233.

FREE, M.J., KAUNE, W.T., PHILLIPS, R.D. & CHENG, H.C. (1981). Endocrinological effects of strong 60 Hz electrical fields in rats. *Bioelectromagnetics*, **2**, 105.

FULTON, J.P., COBB, S., PREBLE, L. *et al.* (1980). Electrical wiring configurations and childhood leukaemia in Rhode Island. *Am. J. Epidemiol.*, **111**, 292.

INOUE, M., MILLER, M.W., COX, C., & CARSTEEN, E.L. (1985). Growth rate and mitotic index analysis of *Vicia faba* L roots exposed to 60 Hz electrical fields. *Bioelectromagnetics*, **6**, 293.

MCDOWALL, M.E. (1986). Mortality of Persons Resident in the Vicinity of electricity Transmission Facilities. *Br. J. Cancer*, **53**, 271.

MYERS, A., CARTWRIGHT, R.A., BONNELL, J.A. & CARTWRIGHT, S.C. (1985). *Overhead Power Lines and Childhood Cancer*. IEE Conference, Publication 257, p.118

NATIONAL RADIOLOGICAL PROTECTION BOARD (1989). *Document GS11 (Guidance on Standards)*. Guidance as to restrictions on exposures to time varying electromagnetic fields and the 1988 recommendations of the International Non-ionizing Radiation Committee. NRPB: Didcot.

PHILLIPS, J.L., WINTERS, W.D. & RUTLEDGE, L. (1986). *In vitro* exposure to electromagnetic fields: changes in tumor cell properties. *Int. J. Radiat. Biol.*,.**49**, 463.

SAVITZ, D.A., WACHTEL, H., BARNES, F.A. *et al.* (1988). Case-control study of childhood cancer and exposure to 60-Hz magnetic fields. *Am. J. Epidemiol.*, **128**, 21.

SEVERSON, R.K., STEVENS, R.G., KAUNE, W.T. *et al.*, (1988). Acute nonlymphocytic leukemia and residential exposure to power frequency magnetic fields. *Am. J. Epidemiol.*, **128**, 10

TOMENIUS, L. (1986). 50-Hz electromagnetic environment and the incidence of childhood tumors in stockholm county. *Bioelectromagnetics*, **7**, 191.

WERTHEIMER, N. & LEEPER, E. (1979). Electrical wiring configurations and childhood cancer. *Am. Epidemiol.*, **109**, 273.

WERTHEIMER, N. & LEEPER, E. (1980). Electrical wiring configurations and childhood leukaemia in Rhode Island. *Am. J. Epidemiol.*, **111**, 461.